# An EST screen from the annelid *Pomatoceros lamarckii *reveals patterns of gene loss and gain in animals

**DOI:** 10.1186/1471-2148-9-240

**Published:** 2009-09-25

**Authors:** Tokiharu Takahashi, Carmel McDougall, Jolyon Troscianko, Wei-Chung Chen, Ahamarshan Jayaraman-Nagarajan, Sebastian M Shimeld, David EK Ferrier

**Affiliations:** 1 Faculty of Life Sciences, University of Manchester, Oxford Road, Manchester, UK; 2 The Scottish Oceans Institute, University of St. Andrews, St. Andrews, Fife, UK; 3 Centre for Ornithology, School of Biosciences, University of Birmingham, Edgbaston, Birmingham, UK; 4 Department of Zoology, University of Oxford, South Parks Road, Oxford, UK; 5 Department of Biochemistry, University of Oxford, South Parks Road, Oxford, UK; 6 School of Biological Sciences, University of Queensland, St Lucia, Queensland, Australia

## Abstract

**Background:**

Since the drastic reorganisation of the phylogeny of the animal kingdom into three major clades of bilaterians; Ecdysozoa, Lophotrochozoa and Deuterostomia, it became glaringly obvious that the selection of model systems with extensive molecular resources was heavily biased towards only two of these three clades, namely the Ecdysozoa and Deuterostomia. Increasing efforts have been put towards redressing this imbalance in recent years, and one of the principal phyla in the vanguard of this endeavour is the Annelida.

**Results:**

In the context of this effort we here report our characterisation of an Expressed Sequence Tag (EST) screen in the serpulid annelid, *Pomatoceros lamarckii*. We have sequenced over 5,000 ESTs which consolidate into over 2,000 sequences (clusters and singletons). These sequences are used to build phylogenetic trees to estimate relative branch lengths amongst different taxa and, by comparison to genomic data from other animals, patterns of gene retention and loss are deduced.

**Conclusion:**

The molecular phylogenetic trees including the *P. lamarckii *sequences extend early observations that polychaetes tend to have relatively short branches in such trees, and hence are useful taxa with which to reconstruct gene family evolution. Also, with the availability of lophotrochozoan data such as that of *P. lamarckii*, it is now possible to make much more accurate reconstructions of the gene complement of the ancestor of the bilaterians than was previously possible from comparisons of ecdysozoan and deuterostome genomes to non-bilaterian outgroups. It is clear that the traditional molecular model systems for protostomes (e.g. *Drosophila melanogaster *and *Caenorhabditis elegans*), which are restricted to the Ecdysozoa, have undergone extensive gene loss during evolution. These ecdysozoan systems, in terms of gene content, are thus more derived from the bilaterian ancestral condition than lophotrochozoan systems like the polychaetes, and thus cannot be used as good, general representatives of protostome genomes. Currently sequenced insect and nematode genomes are less suitable models for deducing bilaterian ancestral states than lophotrochozoan genomes, despite the array of powerful genetic and mechanistic manipulation techniques in these ecdysozoans. A distinct category of genes that includes those present in non-bilaterians and lophotrochozoans, but which are absent from ecdysozoans and deuterostomes, highlights the need for further lophotrochozoan data to gain a more complete understanding of the gene complement of the bilaterian ancestor.

## Background

*Pomatoceros lamarckii *is a tube-building serpulid annelid. The hard, calcareous tubes are common features on hard substrata such as rocks, marine infrastructure, molluscan shells and large crustacean exoskeletons. As such they can become important biofouling agents as well as being a concern to the aquaculture industry, for example, mussel farming. *P. lamarckii *has also proven to be a useful model in marine ecotoxicology, being widespread in the intertidal and sub-littoral zones (along with its sister species *Pomatoceros triqueter*) with easily accessible embryonic and larval material [1] as well as a distinctive karyotype amenable to evaluation of gross chromosomal aberrations and changes [2,3].

As an annelid *P. lamarckii *is a member of the Lophotrochozoa clade, or super-phylum, of bilaterian animals. The lophotrochozoan clade is still relatively poorly represented in terms of genomic resources compared to the other two major clades of bilaterians; the Ecdysozoa (containing arthropods, nematodes and other related phyla) and the Deuterostomia (chordates, echinoderms and hemichordates) [4]. This imbalance needs to be redressed in order to obtain a more balanced understanding of animal genome evolution and comparative biology.

Coral ESTs [5] and the *Nematostella vectensis *genome [6], and to a lesser extent the genome of *Trichoplax adhaerens *[7], have revealed a surprisingly high level of gene conservation between radiate, or diploblast, animals and bilaterians, in terms of numbers of genes, gene family representation and gene organisation. This level of surprise has to a certain extent been due to the erroneous estimated levels of gene conservation across the Bilateria due to the bias in sampling from mainly Deuterostomia and Ecdysozoa. The coral EST data [5] in particular revealed that many genes that had previously been thought to be deuterostome or vertebrate innovations, are in fact conserved with this non-bilaterian animal and hence have been lost in the lineages leading to the ecdysozoan genomes sampled. To truly understand these patterns of lineage and clade-specific gene losses and conservation, a broader phylogenetic sample of taxa must be compared. Although the recent diploblast/Radiata to triploblast/Bilateria comparisons have clearly revised our concept of the complexity (in genetic terms) of the last common ancestor of these groups, there is still a huge amount to be determined about the major events in animal evolution at the transition between these two groups, and about the nature of the last common ancestor of the Bilateria. Reconstructing the gene catalogue or complement of this ancestor, from which 99% (in terms of species number) of the extant animal kingdom subsequently evolved is a crucial goal for understanding animal evolution.

Of the handful of Annelida that do have collections of gene sequences available, most data-sets tend to have been produced from material exposed to specific conditions or protocols (e.g. subtractive hybridisations [8,9]) and hence whilst they give extremely valuable information about those specific processes they do not necessarily provide enough information for obtaining a more general picture of bilaterian evolutionary genomics. For those annelids that do have more extensive sequence data available [10], or in production [11,12], a preliminary picture is emerging of the great potential utility of polychaetes for comparative biology, since species like *Platynereis dumerilii *appear to have retained more of the ancestral characters (genes and networks) from the bilaterian ancestor than other protostome model systems [10,13,14]. *Pomatoceros *provides a useful complement to annelids such as *Platynereis *with regard to its life history, morphology and potentially its phylogenetic position. *Pomatoceros *is sedentary, living in a permanent, fixed tube, whilst *Platynereis *can be considered an errant annelid, moving around its benthic habitat. *Pomatoceros *has a heteronomous body form, with a tentacular crown and distinctive head, thorax and abdominal regions, whilst *Platynereis *is more homonomous, with a head and more uniform trunk. With regard to phylogeny *Pomatoceros *and *Platynereis *are considered as members of two of the major divisions of polychaetes, the Aciculata (includes *Platynereis*) and the Canalipalpata (includes *Pomatoceros*) [15]. However, recent molecular phylogenetic data highlights the fact that the family-level phylogeny of the annelids is rather poorly resolved and the Aciculata and Canalipalpata may not be bona fide groupings [16]. Clearly, a wider sampling of taxa is essential to test the general utility of polychaetes, or whether the "less-derived" characteristics of species such as *P. dumerilii *are more restricted to specific taxa.

More widely in the Lophotrochozoa whole genome sequences are now available for a very small number of taxa, and EST data is also available for a handful of species [17-20]. As this type of data increases further we will be able to expand and refine the types of analyses performed and obtain a much clearer picture of the patterns of gene and gene family conservation, gain and loss across the bilaterians, and hence the gene complement of the bilaterian ancestor from which the vast majority of present-day animals evolved.

Here we report the characterization of over 5,000 ESTs from the annelid *P. lamarckii*, and make detailed comparisons of these ESTs with data from across the animal kingdom. We find that *P. lamarckii *exhibits relatively short branches in molecular phylogenetic trees, permitting clearer, more robust comparisons to genes from other taxa than is possible with very long-branch organisms such as *Caenorhabditis elegans *and *Ciona intestinalis*. We have classified the *P. lamarckii *sequences that have orthologues in other animals into categories that reveal patterns of gene loss from the Ecdysozoa and/or Deuterostomia. It is clear that the ecdysozoan genomes sampled to date have undergone a significant amount of gene loss relative to the other bilaterian clades. If we are to understand the function of these genes we cannot rely on the more traditional protostome model systems of insects and nematodes from which these genes have been lost. As well as genes that are widely conserved across two or three of the major bilaterian clades we find a class of genes that is ancient in the animals, being conserved with non-bilaterian animals, but which appears to have been retained only in a single bilaterian super-phylum, in this case the Lophotrochozoa. Such genes may reveal specific aspects of lophotrochozoan biology. A further class of genes that are restricted to only lophotrochozoan animals are also promising candidates for understanding the evolution and biology of this important group of animals, and may provide candidates for exploitation for resolving the phylogeny of the lophotrochozoan phyla.

## Results and Discussion

### A collection of larval genes in *P. lamarckii*: assembly statistics

A total of 5664 ESTs were generated from a non-normalised cDNA library of *P. lamarckii *larvae. These sequences are available under GenBank accession numbers GR308222 to GR312353. The ProcessEST in-house pipeline programme denoted 1794 of these as singletons, and clustered the remainder to form 521 contigs. Singletons have a mean length of 655 bp, whilst contigs have a mean length of 967 bp. Of the 42 clusters containing 10 or more EST individual sequences, the majority code for ribosomal proteins, together with house-keeping genes such as elongation factor 1-alpha (see Additional file 1). However, 6 clusters do not yield significant matches to any other proteins or ribosomal RNA in any database at all. They consist of at least 12 ESTs respectively, indicating that 6 unique sequences are expressed at a high level, when considering the contig for elongation factor 2 (Contig 527) is composed of 12 ESTs. They do not show any matches to registered micro RNA datasets on the miRBase [21] and further work must identify what these sequences code for and why they are expressed at relatively high levels in development of *P. lamarckii*.

GC content averages at 40% across all singletons and contigs, which do not present evidence for genome-wide selection for particularly high or low GC content in *P. lamarckii*. Against half of all ESTs, we conducted a search for start Methionine residues, which yielded 154 ESTs for which it was very likely that the start codon had been reached based on matches to the start site of orthologous proteins along with in-frame stop codons being further 5' to these putative start codons. 5'-untranslated regions (UTRs) have a mean length of 90.0 bp, although these 5'-UTRs are not necessarily all complete due to the way the library was constructed by reverse transcription. Local BLASTn searches within the 5'-UTRs of *P. lamarckii *revealed no evidence for trans-splicing, which has been observed in several other bilaterian phyla [22].

### Shorter *P. lamarckii *branch lengths than 'traditional' invertebrate genomic models

To obtain a general view of *P. lamarckii *gene evolution, we conducted a phylogenetic analysis with 11 other species. These species were chosen due to availability of genome data and also to provide a balanced representation of the three bilaterian super-phyla along with an outgroup, consistent with the latest views of animal phylogeny [23]. A total of 59 orthologous genes were found in all 12 species, forming a concatenated alignment of 11,716 sites, of which 44.2% are informative. See Additional file 2 for a complete list of orthologues and their accession numbers used for this analysis. The concatenated amino acid sequence of a sea anemone (*N. vectensis*) was used in order to root the tree.

**Figure 1:**
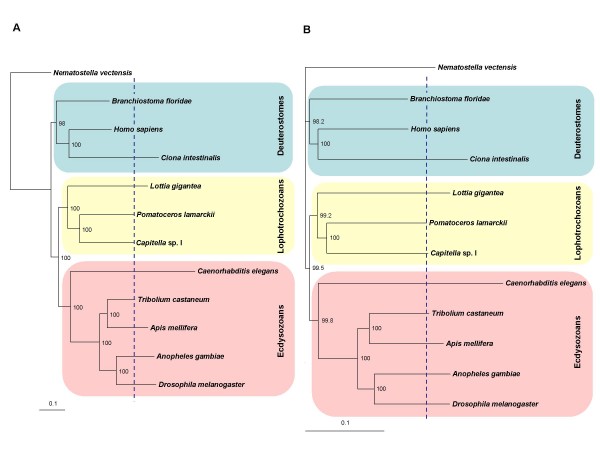
**Phylogenetic trees of concatenated sequences from 11 representative taxa of bilaterian animals**. (A and B) and the non-bilaterian *N. vectensis *as an outgroup (11716 amino acid sites from 59 orthologous genes). The blue vertical dotted line in each of the figures marks the tip of the *Pomatoceros *branch. Bootstrap percentages are indicated on each node. (A) Maximum likelihood tree. Likelihood: Loglk = -138,297.9751. (B) Neighbour-joining tree.

The phylogenetic analysis (Figure 1A and 1B) is noteworthy for two principal things; topology and branch lengths. First, the tree topologies of both the neighbour-joining and the maximum likelihood methods are consistent with the latest views on animal phylogeny of the three clades of the Bilateria [23], with very high support values. The classification of the three super-phyla in the Bilateria, namely the Ecdysozoa, Lophotrochozoa and Deuterostomia, was first proposed by Aguinaldo et al. [4] based on the sequences of small subunit ribosomal RNA. As this view of phylogeny was contradictory to the traditional view of bilaterian phylogeny based mainly on the anatomy of the coelom, the proposal of the three bilaterian clades has often been criticised [24]. However, growing evidence from analyses with datasets from more taxa, and using more appropriate analytical methods, support the three-superphylum phylogeny [23,25]. Our analysis also conforms to the three bilaterian superphylum topology (Figure 1A and 1B). Amongst 11 bilaterian species analysed, 5 species (*Drosophila melanogaster, Anopheles gambiae, Apis mellifera, Tribolium castaneum, C. elegans*) are grouped into one clade corresponding to the Ecdysozoa, 3 species (*P. lamarckii, Capitella *sp. I, *Lottia gigantea*) to the Lophotrochozoa and 3 species (*Branchiostoma floridae, C. intestinalis *and *Homo sapiens*) to the Deuterostomia. It is noteworthy that our trees place *C. elegans *into the Ecdysozoa. Although *C. elegans *is a major model species for invertebrates, it is often excluded from this sort of phylogenetic study to avoid possible tree topology artefacts due to the extremely increased rate of base substitution observed in this species. The long-branch length in our trees reflects the rapid sequence evolution of *C. elegans*, but this has not prevented the resolution of Ecdysozoa in this case. Our trees are also compatible with the recent revision of chordate phylogeny [26,27], in which the cephalochordate, amphioxus (*Branchiostoma*), is now considered to be the basal extant chordate lineage and the urochordates the sister group to the vertebrates. Another noteworthy point is the grouping of *A. mellifera *with *T. castaneum *as sisters. This is contrary to the prevalent view of insect phylogeny in which beetles would be the most basal lineage of the insects represented in our trees [28]. This variability in tree topologies amongst different studies may reflect insect phylogeny being in the process of revision with the input of large molecular datasets in recent phylogenomic studies [29].

Secondly, the branch lengths clearly highlight the significant (P < 0.001) disparity in evolutionary rates between the 'traditional' genomic models of *Drosophila*, *Caenorhabditis *and *Ciona *and other bilaterians (Figure 1A and 1B, and also see Additional file 3 for distance values). By contrast to these model species, polychaetes (*P. lamarckii *and *Capitella *sp. I) are amongst the lineages with some of the shortest branch lengths along with amphioxus (*B. floridae*) in the Deuterostomia and beetle (*T. castaneum*) in the Ecdysozoa. These 'shorter branch length' species, therefore, are likely to be less derived from the ancestral bilaterian condition and so potentially be of greater utility for future phylogenetic and gene evolution analysis than the traditional protostome models of flies and nematodes. We also conducted a similar analysis with a *P. dumerilii *dataset included (unpublished data) and these branch lengths were not significantly different from the trees shown here. Hence, polychaetes as a whole are probably good models for understanding sequence evolution.

### Gene conservation between the three bilaterian super-phyla

**Figure 2:**
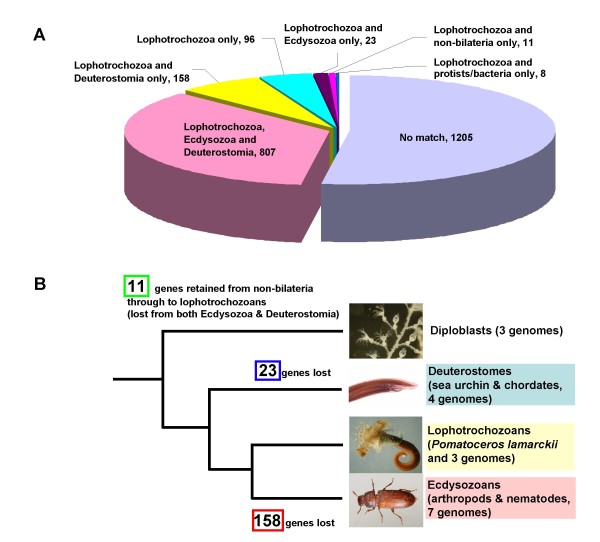
**Summary of the gene conservation analysis**. (A) Distribution of 2308 unique *P. lamarckii *ESTs. Genes were classified into groups based on their pattern of putative orthology with genes in other major metazoan groups. (B) Illustration of gene loss in the evolution of metazoan genomes in reference to *P. lamarckii *orthologues. Refer to Methods for further explanation of classification and gene loss characterization.

Of a total of 2308 EST clusters, 52.2% gave no significant match to other sequences in GenBank or to selected whole genome assemblies (17 animal species) at a BLAST cut-off score of 10^-10 ^(Figure 2A). This percentage is strikingly similar to that recently found in a large-scale EST sequencing project in the hemichordate *Saccoglossus kowalevskii*, where 48% of the unique sequences had no significant match against GenBank when using the same cut-off value [30]. This similarity indicates that the *P. lamarckii *genome is not subject to any pronounced stochastic process that precludes gene identification by similarity searches.

The remaining 47.8% of the EST sequences were divided into classes based upon their putative orthology (by BLAST analysis) to sequences (including genomic information of 17 animal species) in major metazoan groupings. The majority of these (807 genes, 35% of the total EST set) were found in both deuterostomes and ecdysozoans. In addition to genes with general function such as house-keeping genes, this group includes some key genes in animal development that have just recently been shown to be conserved in the Lophotrochozoa as well as the other two groups. For example, orthologues involved in the Notch signalling pathway (pl_xlvo_29e01, Contig 245) [31] or Toll signalling (pl_xlvo_21f05, pl_xlvo_25c05) [32] are found in our collection (See Additional file 4 for all information on gene annotation in this category).

A relatively large proportion (158 genes, 6.8% of the total EST set) was found in lophotrochozoans and deuterostomes only, and not ecdysozoans (Figure 2A and 2B). These genes may well have been lost in the lineage of the Ecdysozoa. By contrast only 23 genes (1.0%) are conserved between lophotrochozoans and ecdysozoans only, and are seemingly absent from deuterostomes. From this contrast of the numbers between these two groups (158 versus 23, Figure 2B), we can infer that gene loss has been more prevalent from the Ecdysozoa than the Deuterostomia. In many analyses, hypotheses about gene evolution only take into account data from a limited range of model animals in either the Deuterostomia or the Ecdysozoa, due to the paucity of information available for the Lophotrochozoa. This analysis indicates that these studies are likely to overestimate the number of novel genes in the Deuterostomia, as ecdysozoans appear to have undergone significant gene loss.

Using the BLAST cut-off score of 10^-10 ^is a rather conservative approach and undoubtedly there will be some orthologues that will be missed by using such a cut-off, particularly for genes with comparatively high evolutionary rates. However, there is no a priori reason to expect that this failure to detect orthologues for 'fast-clock' genes should be biased towards any particular animal super-phylum, since each super-phylum contains 'slow clock' as well as 'fast clock' lineages. Furthermore, use of a more permissive cut-off of 10^-6 ^altered the total numbers of genes in each class, but did not change the overall patterns reported here. Further whole genome sequences, particularly from the Ecdysozoa and Lophotrochozoa, will enable the patterns described here to be refined and the hypothesis of greater levels of gene loss in the Ecdysozoa to be tested even more robustly.

**Figure 3:**
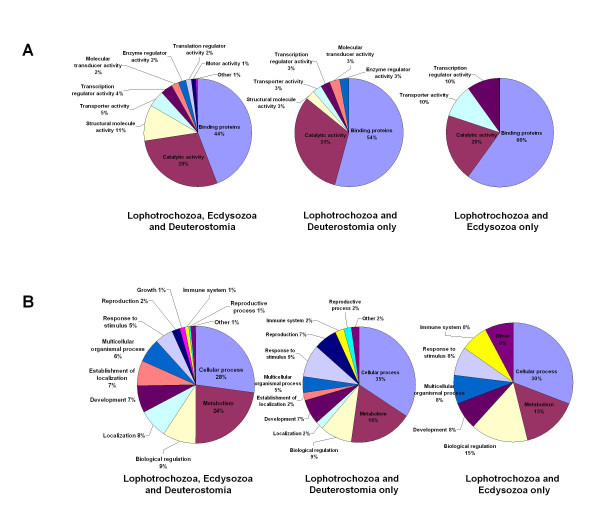
**Comparison of GO annotations associated with EST datasets amongst three major animal groupings after conservation analysis**. (A and B) GO classification according to major GO categories (level 2) by both molecular function (A) and biological process (B) for ESTs classified into three groups, 1) conserved within all three bilaterian clades (807 genes), 2) found only in the Lophotrochozoa and Deuterostomia (lost in the Ecdysozoa, 158 genes) and 3) found only in the Lophotrochozoa and Ecdysozoa (lost from, or never gained by the Deuterostomia, 23 genes).

In this analysis, we chiefly compared the numbers of *P. lamarckii *EST genes in three groups; (1) a group comprising genes conserved in all three bilaterian super-phyla, (2) a group containing genes found only in deuterostomes and lophotrochozoans, which therefore may well have been lost from the Ecdysozoa, and, (3) a group lost from (or never gained by) the Deuterostomia (the "Lophotrochozoa and Ecdysozoa only" group). Figure 3A and 3B show the comparison of Gene Ontology (GO) classifications associated with EST sequences in each of the three groups using characteristics based on mappings to predicted "molecular function" and "biological process". As the numbers of genes and annotated GO terms in each group are varied, it would not be appropriate to draw detailed conclusions directly from this comparison. There are no obvious major biases of GO classification in the types of gene that are being lost, notwithstanding the fact that the 'Lophotrochozoa and Ecdysozoa" class, that represents genes either lost from, or never gained by the Deuterostomia, contains only 23 genes and will inevitably be subject to category sampling error. The possible exception is the genes of the "Structural molecule activity" category, which contains 11% of GO annotations associated with the ESTs conserved in all three super-phyla, but only 3% of GO annotations associated with the ESTs lost from Ecdysozoa (the "Lophotrochozoa and Deuterostomia only" class). Moreover, our statistical analysis using the Fisher's Exact Test showed that the genes related to the "Structural molecule activity" are significantly overrepresented in the group conserved in all three super-phyla, and underrepresented in the other two groups against the whole dataset (adjusted p-value < 0.01). Perhaps genes related to structural function are less likely to be lost during animal evolution. Details of genes in each of the three groups and their annotation are found in Additional files 4, 5 and 6.

Interestingly, GO annotations in the 'reproduction' category contribute 2% of the GO annotations for the genes conserved within all the three super-phyla but 7% of the GO annotations conserved between the "Lophotrochozoa and Deuterostome only" (none of these reproduction GO annotations are found in the group conserved in the "Lophotrochozoa and Ecdysozoa only"). Perhaps the genes in this category tend to be more easily lost particularly from the ecdysozoan lineage: this observation is statistically supported (underrepresented in the "Lophotrochozoa and Ecdysozoa only", adjusted p-value < 0.01). The *Pomatoceros *reproductive genes of which orthologues are not found in any ecdysozoan genomes but which are conserved with the Deuterostomia include; sperm flagellar 2 protein (pl_xlvo_03f08), spermatogenesis associated 4 protein (pl_xlvo_13g04), spermatogenesis serine-rich 1 protein (pl_xlvo_14h03), sperm associated antigen 17 protein (pl_xlvo_21e04) and spermatogenesis associated 18 protein (pl_xlvo_21h03). Little is known about the evolutionary origin of spermatogenesis as the majority of research on spermatogenesis is limited to mammalian systems. Spermatogenesis research outside vertebrates is primarily conducted on *Drosophila*. Although some similarity of *Drosophila *spermatogenesis to mammals is already known [33], our data suggest that molecular mechanisms underlying spermatogenesis have possibly been more modified in the lineage of the Ecdysozoa, including *Drosophila*, than in the Lophotrochozoa. It may well be fruitful to investigate the molecular genetics of spermatogenesis in annelids, to gain a more robust picture of the extent of conserved mechanisms between protostomes and deuterostomes. Also, in relation to these 'reproduction' genes, a *Pomatoceros *orthologue of vertebrate placenta-specific 8 protein is present in the "Lophotrochozoa and Deuterostomia only" group. Although we do not know the functional properties of this protein, this is one of the most highly expressed genes in our EST collection (Contig 530 includes 12 ESTs and Contig 194 contains 2 ESTs; the separation into 2 contigs is due to polymorphisms), suggesting a prominent role in an early larval biology process in *P. lamarckii*.

The number of the genes from our dataset that have been lost from the Ecdysozoa is 158, far more than the 23 genes lost from the Deuterostomia. Together with the phylogenetic analysis discussed in the previous section, this result indicates that ecdysozoan genomes have undergone more modifications during evolution, and as a result, are more divergent from, and have lost more genes than those of the two other super-phyla in the Bilateria, namely the Lophotrochozoa and Deuterostomia. As our analyses are based on sequences of lophotrochozoans, primarily *P. lamarckii*, we could not estimate the number of the genes that might have been lost in the lineage of the Lophotrochozoa from our data. However, in a study using a cnidarian EST dataset, a similar conclusion of extensive ecdysozoan gene loss has also been suggested [5]. The study compared 1376 ESTs of a sea coral *Acropora millepora *with genomic data from *D. melanogaster*, *C. elegans *and *H. sapiens*. Since this coral EST report, more molecular data from a greater variety of taxa and several more genome projects have become available. We included the latest information available here (for example, genomic information of 5 species in the Ecdysozoa including a crustacean *Daphnia pulex*). We can, therefore, infer with more confidence that extensive gene loss may have occurred not only in the model ecdysozoans such as *D. melanogaster *and *C. elegans *but could be a more general event for the Ecdysozoa as a whole. However, the majority of genomic data is still biased towards two major groups in the Ecdysozoa, nematodes and arthropods, and we need genomic information from further ecdysozoan phyla (such as priapulids) to enable us to draw this conclusion with even greater confidence.

One of the major differences from the previous cnidarian EST work is that because our analysis is based on sequences of the Lophotrochozoa, it provides more precise information about the gene loss/gain situation within the Bilateria. From comparison between the Cnidaria and the three model animals in the Deuterostomia and Ecdysozoa, it is impossible to decide whether the genes that are uniquely found in deuterostomes are novel genes in the Deuterostomia, or they were already present in a common metazoan ancestor but were subsequently lost in the Cnidaria as well as the Ecdysozoa. Inclusion of lophotrochozoan data, as we have done here, provides much greater resolution as to the genes that are, and are not, deuterostome-specific and so have been changed on the lineage leading to ourselves. A good example is the thyroid hormone system. It was originally thought to be a unique system in chordates as the genes related to this system had not been found elsewhere [34]. Indeed the amphioxus thyroid system has recently been shown to induce metamorphosis [35], suggesting that mechanisms underlying metamorphosis are conserved between protochordates and vertebrates. When the related genes were then found in non-chordate animals, including the lophotrochozoan *Aplysia *[36], the evolutionary origin of the genetic basis of this important hormonal system was realised to be older than previously thought. In our study, we also detected a *Pomatoceros *gene orthologous to the chordate iodothyronine deiodinase gene (pl_xlvo_36e12). This EST encodes the downstream region of a domain known to be important for vertebrate deiodinase function [37], but further work to isolate more upstream sequence of this gene will be required to determine whether this gene encodes an active deiodinase. In any case, functional analysis of these genes in lophotrochozoans will facilitate our understanding of the evolutionary origin of the thyroid system as well as metamorphosis.

### Metazoan genes lost from both Deuterostomia and Ecdysozoa

Reconstructing the gene complements, or repertoires, of the last common ancestor of the Bilateria and Metazoa are important goals for evolutionary biology, due to these ancestors having been the starting points for the major diversifications of the animal kingdom. Several recent whole genome scale studies [6,7,38] suggest that ancient animal genomes may have been more complicated than originally thought, with previous misconceptions arising from limited taxon sampling that was largely restricted to a small handful of (often derived) model organisms. In this regard, lophotrochozoan data such as our *P. lamarckii *EST dataset can reveal genes that originated in the common ancestor of the Metazoa and were retained into the bilaterian ancestor, but have been lost in both the Deuterostomia and Ecdysozoa, and were therefore previously regarded as genes specific to non-bilaterian animals.

By comparison of our data with available genomes, including three non-bilaterian species [6,7,38], 11 genes appear to be exclusively shared between non-bilaterians (the Cnidaria, Placozoa and Porifera) and *P. lamarckii*, but have been lost in all other bilaterian taxa so far examined. These 11 ESTs were fully sequenced and further characterised since these genes have the potential to reveal particular aspects of lophotrochozoan and bilaterian ancestor biology that cannot be extrapolated from the traditional model systems in the Ecdysozoa and Deuterostomia. The information gathered for each gene is summarised in Table 1. Seven of these genes are conserved with cnidarians (*Acropora*, *Nematostella *and *Hydra*) (see Table 1). Two ESTs (pl_xlvo_44b10 [GenBank: GQ381301] and pl_xlvo_47g05 [GenBank: GQ381303]) are conserved even with the unicellular choanoflagellate *Monosiga *which is a protist lineage relatively closely related to the Metazoa. The remaining 4 genes have been lost even in the cnidarian genomes sampled, and are only conserved with placozoan or sponge genomes. From these latter 4 genes, Contig 270 [GenBank: GQ381302] and 381 [GenBank: GQ381305] are similar to each other but are in fact different genes (42.4% similarity at the amino acid level).

**Table 1:** Characterisation of lophotrochozoan/non-bilaterian specific genes.

EST	Information from BLAST match	Domains	Cnidaria	Placozoa	Porifera
pl_xlvo_20b10	Nucleoporin 205 kDa	None predicted	**+**	**-**	**-**

pl_xlvo_22d01	Only matches predicted proteins	None predicted	**+**	**-**	**-**

pl_xlvo_24g08 (Contig86)	Calcium dependent mitochondrial carrier protein	EF-hand calcium binding domain (two domains), flagellar calcium binding domain, possibly involved in motility	**+**	**-**	**-**

pl_xlvo_26e03	MAR-binding filament-like protein 1 from plants, similar to IQ motif containing E from zebrafish	IQ motif - binding site for EF-hand proteins	**-**	**+**	**-**

pl_xlvo_39g10	Lactoylglutathione lyase from plants, glutathione S-transferase from anemone	Soluble glutathione S-transferase C-terminal domain - detoxification of reactive electrophilic compounds and in metabolite biosynthesis	**+**	**-**	**-**

pl_xlvo_43h12 (Contig163)	Membrane-associated protein from cyanobacteria	Methyltransferase type 11 - modifies DNA, RNA, proteins and small molecules for regulatory purposes	**-**	**+**	**-**

pl_xlvo_44b10	Endonuclease I	Endonuclease I, Bacterial, periplasmic. Generates double stranded breaks in DNA.	**+**	**-**	**-**

pl_xlvo_46h11 (Contig270)	Regulatory P-domain containing protein	Uncharacterised conserved protein	**-**	**-**	**+**

pl_xlvo_47g05	Putative alpha-amylase from slime mould	Glycosyl hydrolase, family 13, catalytic region - hydrolyse the glycosidic bond between two or more carbohydrates, or between a carbohydrate and a non-carbohydrate moiety	**+**	**+**	**+**

pl_xlvo_53g01	Apextrin from coral	Kringle domain - thought to play a role in binding mediators, such as membranes, other proteins or phospholipids, and in the regulation of proteolytic activity Membrane attack complex components/perforin signature - forms transmembrane channels which disrupt the phospholipid bilayer of target cells, leading to cell lysis and death	**+**	**-**	**-**

pl_xlvo_56c06 (Contig381)	Regulatory P-domain containing protein	WD40 repeat - signal transduction, transcriptional control, cell-cycle control and regulation of apoptosis	**-**	**-**	**+**

Domain prediction and descriptions are from INTERPROSCAN and/or PROSITE. Three columns at right side show presence/absence (+/-) of orthologue for each EST in non-bilaterian genomes searched. The entire sequences of these ESTs are available under GenBank accession numbers GQ381295 to GQ381305.

Of the 11 genes, four appear to have an enzymatic function, two are presumably involved in calcium signalling, one has similarity to apextrin (a cnidarian developmental gene) [GenBank: ABK63971], one has similarity to nucleoporin and therefore appears to be a structural protein, and three are completely uncharacterised. Apextrin is also found in echinoderms and in amphioxus, therefore if pl_xlvo_53g01 [GenBank: GQ381304] is an apextrin orthlogue, it is also found in non-lophotrochozoan bilaterians and should be discounted from the lophotrochozoan/non-bilaterian group. Interestingly, pl_xlvo_53g01 also contains a kringle domain [Pfam:PF00051], which is absent from all other apextrins, and places doubt as to the orthology assignment to apextrin. It is likely that pl_xlvo_53g01 is in fact a distinct protein and only matches apextrin in BLAST searches due to the MAC domain [Pfam:PF01823] which is shared between these genes. This example demonstrates that the methods utilised to assign orthology in large scale EST surveys such as ours (i.e., similarity searching by the BLAST algorithm) are susceptible to error and can only be used to obtain a broad idea of the biology of *P. lamarckii*, more precise assessments of orthology require further, focused sequencing and individual assessment of each gene.

Of the 11 genes that are shared between *P. lamarckii *and non-bilaterian animals, 6 are conserved with other lophotrochozoan animals, *Capitella *and/or *Lottia*. In a study analogous to our analysis, Moroz and colleagues used an *Aplysia *EST dataset, and reported 20 genes that were uniquely shared between the cnidarian *Hydra *and *Aplysia *[19]. One specific example was the universal stress protein superfamily [Pfam:PF00582; COGs:COG0589] that had been thought to be limited to the Archaea, Eubacteria and plants. Following the discovery of cnidarian orthologues [39], Moroz and colleagues found *Aplysia *orthologues and argued that such genes have been lost from the other two bilaterian super-phyla. We also found a *P. lamarckii *orthologue (Contig 136). However, in our analysis we classified this gene into the group of the genes that are lost in Ecdysozoa ("Lophotrochozoa and Deuterostomia only") since we found some orthologues in a *Ciona *genome [GenBank: XP_002130444, XP_002122447, XP_002130756, XP_002130652, XP_002130531, XP_002130913, XP_002119695, XP_002130688]. As this example shows, this type of analysis benefits from more data from a wider variety of species, and our results could be revised after more information becomes available. Nonetheless, some of these genes could be keys to understand unique features of lophotrochozoan biology, as well as aspects of the bilaterian ancestor that cannot be inferred from traditional ecdysozoan and deuterostome model systems.

### *Pomatoceros *genes restricted to lophotrochozoans

The Lophotrochozoa are not only an important clade for understanding the ancestry of metazoans and bilaterians, but are also themselves an extremely diverse group of animals exhibiting much evolutionary change and novelty which is likely to involve, at least to some extent, lophotrochozoan-specific genes. We found that 96 *Pomatoceros *ESTs are uniquely conserved within the Lophotrochozoa only. The genes classified into this group are shared between *P. lamarckii *and at least one more species in the Lophotrochozoa. Amongst the 96 genes, 62 genes are conserved only in annelids (e.g., *Capitella *sp. I or *Helobdella robusta*). Since most BLAST matches in this Lophotrochozoa-restricted group are to merely computationally predicted proteins, we do not have much information about their biological function at present. Thus we analysed functional domains and cellular localisation using the computer programmes, INTERPROSCAN, CDD and PSORT. The results are summarised in Additional file 7. This list of the genes will provide a basis for future molecular analysis of *P. lamarckii*, annelids and the Lophotrochozoa.

Although functional characterisation and annotation for most of the genes in this category requires future analysis, the biological properties of a small number (7 genes) have been functionally analysed in other lophotrochozoan species, and they predominantly have neuromuscular roles. All seven of them show a high level of similarity at the protein level and their function is likely to be conserved in *Pomatoceros*. Table 2 lists the *P. lamarckii *ESTs with functions characterised in other lophotrochozoan taxa.

**Table 2:** Characterisation of 7 *Pomatoceros *genes whose putative function is deduced by extrapolation from other lophotrochozoans.

EST	Information from BLAST match	Domains	Inferred function
pl_xlvo_23a09	PRQFVamide precursor protein from *Aplysia*	The predicted --FV amide neuropeptide in annelids, signal peptide	Regulation of the feeding system

pl_xlvo_33f01 pl_xlvo_58g09	29-kDa galactose-binding lectin from *Lumbricus*	IPR000772, IPR008997 Ricin B lectin, cd00161, Ricin-type beta-trefoil, signal peptide	the axon targeting in the central nervous system

pl_xlvo_42f05	Cerebrin prohormone from *Aplysia*	The predicted first annelid Cerebrin, two dibasic cleavage sites, signal peptide	the feeding motor pattern

Contig 102	N/A	IPR002048 Calcium-binding EF-hand, IPR012619 Myoactive tetradecapeptides	Contractions of the gut muscles

Contig 196	FMRFamide precursor protein from *Lymnaea*	The neuropeptide Phe-Met-Arg-Phe-NH2 (FMRFamide) is a potent cardioactive neuropeptide in *Lymnaea stagnalis*	Cardioactive neuropeptide

pl_xlvo_55b08 (Contig 84)	Dopamine beta hydroxylase-like protein from *Aplysia*	A unique structure in lophotrochozoans, with combination of temptin at the N-terminal and two monooxygenase domains at the C-terminal	Pheromone and monoxygenase

Domain prediction and descriptions are from INTERPROSCAN. Inferred functions are from other studies (see text for details).

EST (pl_xlvo_23a09) shows high similarity to an *Aplysia *PRQFVamide precursor protein [GenBank: AAO73964]. The PRQFVamide was identified as a novel pentapeptide from *Aplysia *[40], expressed in the central nervous system and gut. It suppresses contraction of the gut and vasculature and is suggested to act as a modulator within the feeding system. Interestingly, pl_xlvo_23a09 does not have a repeat of the exact PRQFV motif identified in *Aplysia*. Instead, its repeated motif has a deletion of a glutamine at the third position or it has other types of -FV amide motif that are shared with the three neuropeptide families in *Aplysia *such as AMRPs [41] and enterins [42]. We therefore completely sequenced the clone from which the original EST was derived and obtained a provisional full-length cDNA [GenBank: GQ381306] (see Additional file 8). It is still difficult to determine the correspondence of this gene to the three *Aplysia *precursors of -FV amide neuropeptides, however, the neuropeptides from this *Pomatoceros *gene are likely to act on the gut in *Pomatoceros *as well, since all three families of *Aplysia *-FV amide neuropeptides have a similar function.

Two EST clones (pl_xlvo_33f01 and pl_xlvo_58g09) are well conserved amongst annelids, *P. lamarckii, Capitella *sp. I and *H. robusta*. These two genes have related sequence motifs, but encode two different proteins (only 19.4% similarity at the amino acid level). And both show the same top match to the *Lumbricus *29-kDa galactose-binding lectin [GenBank: BAA36393] [43] that was originally identified as a galactose-specific lectin. The top BLAST match, as well as the outcome of INTERPROSCAN and CDD, indicated that these predicted proteins are *Pomatoceros *galactose-binding lectins with the Ricin B lectin motifs [InterPro:IPR000772, IPR008997; CDD:cd00161]. This identification was confirmed by completion of the entire EST clone sequence of 58g09, which provided a probable full-length cDNA [GenBank: GQ381307] (see Additional file 8).

**Figure 4:**
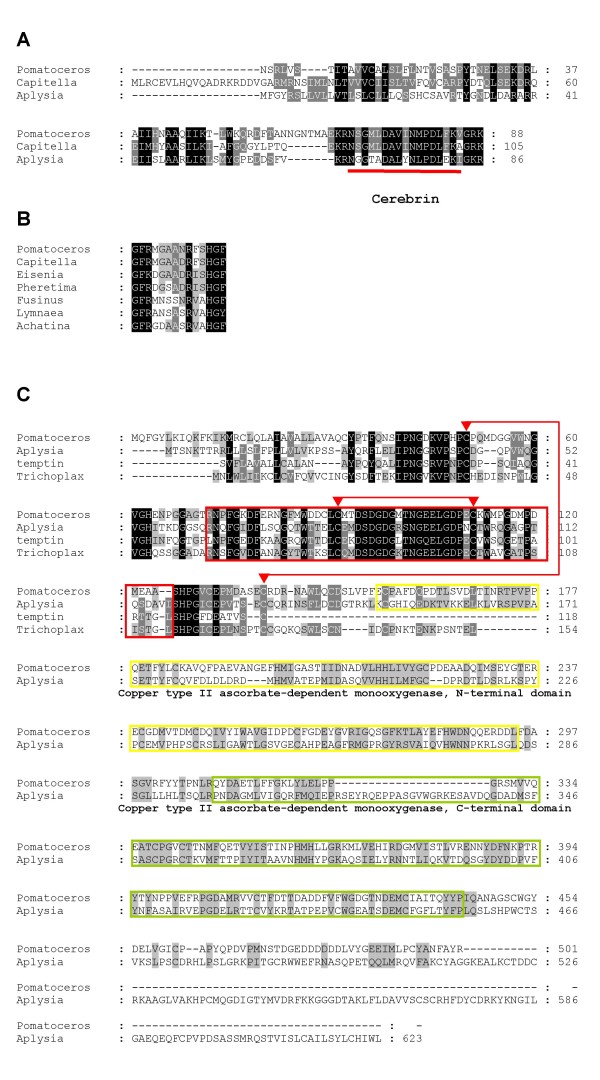
**Amino acid alignments of the proteins with putative lophotrochozoan-specific functions**. (A) Predicted proteins from *Pomatoceros *EST clone (pl_xlvo_42f05) and the *Capitella *genome are aligned with *Aplysia *cerebrin prohormone. The 17-residues of the cerebrin amidated peptide purified from *Aplysia *is underlined. (B) Alignment of tetradecapeptide sequences from 4 annelid and 3 mollusc species with 2 predicted sequences from a *Pomatoceros *EST (Contig 102) and the *Capitella *genome. (C) Comparison of the *Pomatoceros *protein predicted from EST clone (pl_xlvo_55b08) with *Aplysia *dopamine beta hydroxylase-like protein, *Aplysia *temptin protein and a hypothetical protein predicted from the *Trichoplax *genome. The central 1 region of temptin is boxed with red lines. Two disulfide bonds are marked with red lines and triangles. The conserved regions of mono-oxygenase are also boxed with yellow (N-terminal domain) and green (C-terminal) lines respectively.

The EST pl_xlvo_42f05 is highly similar to a *Capitella *gene [JGI:jgi|Capca1|204689|fgenesh1_pg.C_scaffold_17000004] that shows a unique match to cerebrin prohormone [GenBank: AAM00268], which has a profound effect on the feeding motor pattern in *Aplysia *[44]. Despite its remarkable effect on the feeding motor pattern, there are no similar peptides reported outside gastropods; however, as both annelid genes encode a highly conserved region corresponding to the 17-residue peptides of cerebrin as well as two dibasic cleavage sites (Figure 4A), it is very likely that these genes encode *Pomatoceros *and *Capitella *orthologues of *Aplysia *cerebrin prohormone.

Although a clustered sequence (Contig 102) does not show any significant BLAST matches, apart from a *Capitella *predicted sequence [JGI:jgi|Capca1|226681|estExt_fgenesh1_pg.C_470016], the INTERPRO analysis predicted that the protein encoded by this gene includes amino acid sequences that appear orthologous to the myoactive tetradecapeptide family isolated from the gut of earthworms, *Eisenia foetida *and *Pheretima vitata *[45] [InterPro:IPR012619]. The peptides of both earthworm species have a potent excitatory action on spontaneous contractions of the anterior gut. Similar tetradecapeptides have also been purified from molluscan lophotrochozoans [46,47] and have either excitatory or inhibitory effects on muscles, mainly of the gastrointestinal tract. Figure 4B shows an alignment of the conserved tetradecapeptide sequences so far reported within the Lophotrochozoa, including the two polychaete sequences identified here. We also found that not only is the tetradecapeptide conserved, but the gene also has an EF-hand calcium-binding domain at the C-terminal. To our knowledge, no animal species outside the lophotrochozoans has this gene.

Contig 196 encodes a partial sequence of a precursor protein containing 4 copies of FMRF motifs (Additional file 8) of which the top BLAST match is the FMRFamide precursor protein of *Lymnaea stagnalis *(great pond snail) [48]. This tetrapeptide was first identified as a cardioexcitatory agent from molluscs [49]. Although it was thought that the distribution of the FMRFamide itself was limited to molluscs, later it was shown that the FMRFamide is a part of a large family of related peptides (FaRPs) and present in every major metazoan phylum examined. The function of the FMRFamide has been extensively studied in molluscs and they are involved in a wide variety of physiological processes such as feeding and reproduction (reviewed by [50]). According to a strict interpretation of our categorisation of genes, on the basis of the 10^-10 ^BLAST cut-off score, the *Pomatoceros *FMRFamide and mollusc genes represent lophotrochozoan-specific genes, and are counted as such in our classification here. However, further work may be required on the classification and phylogeny of these genes and their gene family to understand their phylogeny and taxonomic distribution more clearly, and the relationship between these lophotrochozoan FMRFamide genes and the "extended FMRFamide" genes of insects [51] and the wider family of FaRPs [52].

An entire sequence of one of the EST clones forming the Contig 84 (pl_xlvo_55b08) was determined [GenBank: GQ381308]. It was originally categorised into the group shared with both the Deuterostomia and Ecdysozoa in line with our classification criteria using BLAST; however, we found that this gene encodes a protein with an interesting structure that is only conserved within the Lophotrochozoa (Figure 4C). The predicted protein is clearly an orthologue of *Aplysia californica *dopamine beta hydroxylase-like protein [GenBank: AAY42041] (E-value = 2e-73) that was initially cloned by a large-scale nucleotome study [19]. The C-terminal of this protein is similar to the C-terminal of DBH-like mono-oxygenase proteins that are widely conserved across bilaterians, including two conserved regions of mono-oxygenase [Pfam:PF01082, PF03712, the regions boxed with yellow and green lines respectively in Figure 4C]. The DBH-like mono-oxygenase family has a DOMON domain [Pfam:PF03351] in common at the N-terminal. In contrast, the N-terminal of both the *Pomatoceros *and *Aplysia *proteins are only conserved with a different *Aplysia *protein, the pheromone temptin [GenBank: AAS92605] [53] and with a gene predicted from the genome of the basal metazoan lineage *Trichoplax *[GenBank: XP_002117562]. The function and structure of *Aplysia *temptin have been analysed in detail, and the central 1 region of temptin (squared with red lines in Figure 4C) as well as two disulfide bonds (shown by red lines and triangles in Figure 4C) determined by a structure analysis using recombinant temptin [54] are well conserved amongst these genes. We have found that this unique combination of the temptin and mono-oxygenase sequences is also present in the *Lottia *genome [JGI: jgi|Lotgi1|192843|estExt_Genewise1.C_sca_480139] but is not found outside the Lophotrochozoa. This may be a good example of the evolution of new genes by combining genes or exchanging domains, with the result that this novel Temptin/mono-oxygenase 'hybrid' gene is now a lophotrochozoan synapomorphy.

On the basis of our comparative sequence searches we have concluded that this category of "lophotrochozoan-specific genes" is likely to have evolved on the lineage leading to the Lophotrochozoa. An alternative possibility is that at least some of these genes had a more ancient evolutionary origin in the Metazoa, but have then been lost from the non-lophotrochozoan taxa for which genomic data is currently available. Also the genes in this category may have orthologues outside the lophotrochozoans, but we failed to detect them due to high rates of sequence divergence. This is unlikely to apply to all genes in this category however, and so these genes still form a useful starting point for understanding the evolution of lophotrochozoan-specific biology.

Finally, the functions of some of the genes in this group can be predicted from previous studies using other lophotrochozoan species like *Aplysia*. All of these genes with functionally characterized orthologues in *Aplysia *are small peptides that affect neuronal or behaviour activity, such as neuropeptide or mating pheromones. In addition to the genes listed in this group, our EST dataset includes several bioactive small peptides (for example, FVRIamide encoded by Contig 295). Biological effects of these peptides on *Pomatoceros *could potentially be analysed relatively easily via peptide synthesis, without protein purification. Considering the importance of *P. lamarckii *as a biofouling agent in the marine environment, our collection of genes provides a set of candidates that could potentially be used to control *P. lamarckii *populations.

## Conclusion

Comparison of patterns of gene retention across different metazoan groups implies that ecdysozoan genomes, including the established model systems of flies and nematodes, have undergone higher levels of gene loss than other animal groups. Such analysis also provides a clearer picture of the gene complement at major nodes of animal evolution, such as the origin of the Metazoa, the origin of the Bilateria, and the origin of the Lophotrochozoa. Molecular phylogenetic analyses demonstrate the usefulness of annelids for evolutionary study as less divergent species from these ancestral states than several, more traditional, invertebrate model systems. Our EST analysis also highlights the potential for the rapid isolation of evolutionary novel genes at various evolutionary and taxonomic levels.

## Methods

### Animal collection and larval culture

Adult *P. lamarckii *worms were collected from the littoral zone at Tinside, Plymouth, UK, and were maintained at 12°C in a recirculating aquarium system. Adult worms were removed from tubes by breaking open the posterior section and forcing the animals backwards using a blunt probe. Fertile animals began releasing gametes at this stage. Male and female worms were immediately transferred to separate Petri dishes containing filtered, UV treated seawater (FSW). Eggs were rinsed through a 100 um cell strainer (Falcon) to remove tube debris, and then collected in a 40 um cell strainer (Falcon). The strainer was then transferred to a Petri dish containing a dilute sperm suspension, agitated, and left for 10 minutes to allow fertilisation to occur. Eggs were rinsed and gently washed into a 300 mL Pyrex crystallisation dish containing 250 mL FSW. These dishes were covered and incubated at 19°C. *P. lamarckii *trochophore larvae were collected between 24 hours post fertilisation (hpf) and 144 hpf for RNA extraction.

### cDNA library construction

RNA from 24 hpf to 144 hpf larvae of *P. lamarckii *was isolated and a directional cDNA library was constructed by Lofstrand Labs Ltd (Maryland, USA), using the *XhoI *linker primer 5'-GAGAGAGAGAGAGAGAGAGAACTAGTCTCGAG(T_18_) -3'. The *EcoRI *adapters with the sequence 5'-OH-AATTCGGCACGAGG-3' were ligated on to the blunt 5' ends before the library was column purified to remove cDNAs shorter than 400 bp. The cDNA library was then cloned into the *XhoI/EcoRI *site of the Lambda-Zap Express vector (Stratagene, California, USA). The average insert size was 1.05 kb. 1 × 10^6 ^plaque forming units were used as a template to create an amplified library which was then subjected to *in vivo *mass excision of the pBK-CMV phagemid from the Zap Express vector.

### EST isolation, sequencing and classification

After plating of the *P. lamarckii *cDNA library, 5664 independent clones were isolated and sequenced with the T3 vector primer. Raw sequence traces were fed into ProcessEST, an in-house pipeline system developed for the processing and clustering of ESTs. These files are firstly screened for vector contamination and sequence quality using the Phred/Cross_match program [55,56]. The completed EST dataset was also screened manually for vector sequences using local BLASTn searches. This resulted in 567 sequences being discarded. The remaining sequences were then clustered into 2315 EST clusters using Phrap [55,56] (730 bases average length). 1794 ESTs were present as unique sequences, whereas 521 ESTs were represented by multiple clones.

These sequences were then used in a BLASTn analysis for initial identification; seven sequences were identified as ribosomal RNA or *E. coli *contamination and were discarded. The individual sequences constituting these remaining 2308 EST clusters were submitted to GenBank with accession numbers GR308222 to GR312353. For the ESTs constituting the contigs in the text, the related contig ID can be also found in each EST GenBank entry. Several clones were completely sequenced with further sequence-specific oligos (see text for details), and these have GenBank accession numbers GQ381295 - GQ381308.

The 2308 EST clusters were then used in a gapped BLASTx search against the NCBI non-redundant protein sequence database with matches being deemed as significant if the expect threshold was lower than 10^-10^. All significant matches for each EST were then used to classify that sequence into one of several groups depending on the phylogenetic distribution of its identified orthologues. These groups were: no significant matches; found in the Lophotrochozoa, Ecdysozoa and Deuterostomia; found in the Lophotrochozoa and Deuterostomia only; found in the Lophotrochozoa and Ecdysozoa only; found in the Lophotrochozoa only; found in the Lophotrochozoa and non-bilaterians only; found in the Lophotrochozoa and Protista and/or Bacteria only. This was the first stage of our classification against the whole of GenBank, to establish whether there were significant matches to non-animals, non-bilaterian animals, other lophotrochozoans, ecdysozoans and deuterostomes.

This initial classification was further refined by the second-stage of classification against the whole genome databases of the following 16 organisms; *Trichoplax adhaerens, Nematostella vectensis, Capitella *sp. I, *Helobdella robusta, Lottia gigantea, Drosophila melanogaster, Caenorhabditis elegans, Anopheles gambiae, Apis mellifera, Tribolium castaneum, Acyrthosiphon pisum, Daphnia pulex, Strongylocentrotus purpuratus, Branchiostoma floridae, Ciona intestinalis *and *Homo sapiens*. These BLAST searches were conducted on either the Joint Genome Institute (JGI) website [57] or on the NCBI BLAST assembled genomes webpage [58] using BLASTx searches. The ESTs were also submitted to tBLASTx searches of the genomic trace records of *Amphimedon queenslandica *(*Reneira *sp JGI 2005 WGS) and the cnidarian EST dataset. The same cut-off E-value of 10^-10 ^was used in all BLAST searches.

Subsequently all 1103 EST sequences with any significant matches were further analysed for annotation with GO terms and Enzyme codes using Blast2GO [59]. For statistical assessment of GO annotation differences between each subgroup classified by the phylogenetic distribution, the Fisher's exact test with multiple testing corrections (two-tailed) was conducted using the Gossip [60] package implemented in Blast2GO. The whole dataset was used as a reference and Family Wise Error Rate was used as the adjusted p-value of the Fisher's exact test for each GO term.

### Phylogenetic analysis

From the group of genes that are widely conserved amongst the animal kingdom, orthologous genes for use in the phylogenetic analysis were identified from the 11 species: *Nematostella vectensis, Capitella *sp. I, *Lottia gigantea, Drosophila melanogaster, Caenorhabditis elegans, Anopheles gambiae, Apis mellifera, Tribolium castaneum, Branchiostoma floridae, Ciona intestinalis *and *Homo sapiens*. Initially it was determined whether the *P. lamarckii *orthologues demonstrated significant BLASTx matches to orthologous genes in the remaining 11 species. The E-value less than or equal to 10^-10 ^was used as a cut-off value. Where there were multiple paralogous matches within the same species the gene was abandoned unless there was a single, clear orthologue. 59 orthologous genes were extracted from the first half of the EST screen and the protein sequences, together with predicted amino acid sequences from annotated genes, were obtained from the NCBI or JGI gene databases (listed in Additional file 2). The orthologue was not used if it was absent in any of the 11 species selected, resulting in an alignment entirely free of gaps, except for those caused by deletions.

The protein sequences from all 12 species were aligned using CLUSTAL W implemented in the BioEdit software package. The sequences were then trimmed by eye of all sites that were not unambiguously orthologous, and all insertions were removed, unless they were the clear result of a deletion in a minority of the species. The sequences were then concatenated to form one large alignment. The PHYLIP software package [61] was used to create neighbour-joining (NJ) trees using the JTT model of protein evolution with 1000 bootstraps. For maximum likelihood (ML) trees ProtTest 1.4 [62] was used to determine the best protein substitution model and parameters for use in RAxML [63]. The RtREV+I+G+F model of protein evolution was used with the proportion of invariable sites at 0.236014, 4 rates of protein evolution with alpha set at 0.849433, 100 bootstraps were used in the primary ML tree of 12 species. A distance-based relative rate test with RRTree [64] was performed on the concatenated alignment to examine whether relative substitution rates were significantly different between taxa. The concatenated amino acid sequence of *N. vectensis *was used as an outgroup in all the analyses.

### Further analysis of the Lophotrochozoa/non-bilaterians and the Lophotrochozoa only groups

A list of genes that were potentially shared between lophotrochozoans and non-bilaterians (to the exclusion of other bilaterians), was compiled for complete sequencing and further analysis. ESTs were sequenced using specific primers designed approximately 200 bp from the end of the contig together with the T7 primer. Once this sequencing had been finalised, the complete EST sequences were then checked for similarity by BLAST analysis. BLASTp was used when a clear ORF was obvious from translation of EST sequences, BLASTx was used when this was not the case. Each lophotrochozoan specific or lophotrochozoan/non-bilaterian specific EST sequence was then run through INTERPROSCAN [65] and either PROSITE [66] or NCBI Conserved Domains search [67] to search for predicted domains, and also through PSORT [68] in order to predict protein localisation sites.

## Competing interests

The authors declare that they have no competing interests.

## Authors' contributions

TT did the EST sequencing and analyses. CM performed further sequencing and analyses. JT did the initial phylogenetic analyses and W-CC prepared the RNA for the cDNA library. AJ-N scripted the ProcessEST pipeline. SMS and DEKF conceived of the study. DEKF supervised the project and wrote the manuscript with TT. All authors read and approved the final manuscript

## Supplementary Material

Additional file 1 **Distribution of *Pomatoceros *ESTs in the assembled contigs**. The data shows the distribution of *Pomatoceros *EST reads in 521 assembled contigs and the most highly represented putative genes in *P. lamarckii *cDNA library (more than 10 reads). Click here for file

Additional file 2 **A complete list of 59 orthologous proteins that were used for the phylogenetic analysis**. The table lists 59 orthologues and their accession numbers that were used for the phylogenetic analysis. Note that the genes are listed as 60 orthologues because in one case (EEF2) the orthologous ESTs from *P. lamarckii *aligned to discrete regions of this gene. Therefore the two genes are listed as 'a' and 'b' forms. Click here for file

Additional file 3 **A table of the evolutionary distance values in **Figure 1A and 1B. The table shows the evolutionary distance values from the root where *Nematostella *and all bilaterian animal species diverge in Figure 1A and 1B. The exact probability values from the distance-based relative rate tests are also provided. The cells of the table are highlighted with red for significantly higher/longer distance values than that of *Pomatoceros *(P < 0.001) and green for lower/shorter values. Click here for file

Additional file 4 **A complete list of 807 unique *P. lamarckii *EST sequences that are conserved in both the Deuterostomia and the Ecdysozoa**. The table lists 807 unique *P. lamarckii *EST sequences that are significantly (E-value less than 10^-10^) conserved in both the Deuterostomia and the Ecdysozoa. Sequence description, GO terms and Enzyme code are annotated by Blast2GO. Because the BLASTx matches of pl_xlvo_22e07, pl_xlvo_32f10 and Contig 207 are only predicted proteins from genome data, their sequence descriptions and GO terms are not shown here. Click here for file

Additional file 5 **A list of 158 *P. lamarckii *EST sequences that are conserved uniquely with the Deuterostomia**. The table lists 158 *P. lamarckii *ESTs that are conserved uniquely with the Deuterostomia (E-value less than 10^-10^). Sequence description, GO terms and Enzyme code are annotated by Blast2GO. The genes for which sequence descriptions and GO terms are not shown here are those that match only predicted proteins from genome data. Click here for file

Additional file 6 **A list of 23 *P. lamarckii *EST sequences that are conserved uniquely with the Ecdysozoa**. The table lists 23 *P. lamarckii *ESTs that are conserved uniquely with the Ecdysozoa (E-value less than 10^-10^). Sequence description, GO terms and Enzyme code are annotated by Blast2GO. The genes for which sequence descriptions and GO terms are not shown here are those that match only predicted proteins from genome data. Click here for file

Additional file 7 **A list of 96 *P. lamarckii *EST sequences that are conserved only within the Lophotrochozoa**. The table lists 96 *P. lamarckii *ESTs that are conserved only within the Lophotrochozoa (E-value less than 10^-10^). Domain predictions are from INTERPROSCAN and NCBI Conserved Domains searches. Cellular locations were predicted using PSORT. The ESTs highlighted with blue are only conserved within annelids such as *Capitella *and *Helobdella*. Click here for file

Additional file 8 ***P. lamarckii *protein sequences listed in **Table 2. *P. lamarckii *predicted amino acid sequences in Table 2 are provided. The three proteins that are described in Figure 4 are not shown here. A. *Pomatoceros *-FVamide neuropeptide precursor protein sequence predicted from entire sequence of pl_xlvo_23a09 [GenBank: GQ381306]. RPRFV, RRMFV, RPKFV motifs are highlighted with blue. B. *Pomatoceros *galactose-binding lectin sequences predicted from pl_xlvo_33f01 and pl_xlvo_58g09 [GenBank: GQ381307]. The G-X-X-X-Q-X-W motifs are highlighted with yellow and related motif with blue. C. *Pomatoceros *FMRFamide precursor protein sequence predicted from Contig 196. FMRF motifs are highlighted with yellow. Click here for file
