# Supplementary material for: An EST screen from the annelid Pomatoceros lamarckii reveals patterns of gene loss and gain in animals
Source: BMC Evol Biol. 2009 Sep 25;9:240. doi: 10.1186/1471-2148-9-240 (PMC2762978; doi:10.1186/1471-2148-9-240)
Supplement: Additional file 1 — Distribution of Pomatoceros ESTs in the assembled contigs. The data shows the distribution of Pomatoceros EST reads in 521 assembled contigs and the most highly represented putative genes in P. lamarckii cDNA library (more than 10 reads). [file 1471-2148-9-240-S1.PPT]

## Slide 1
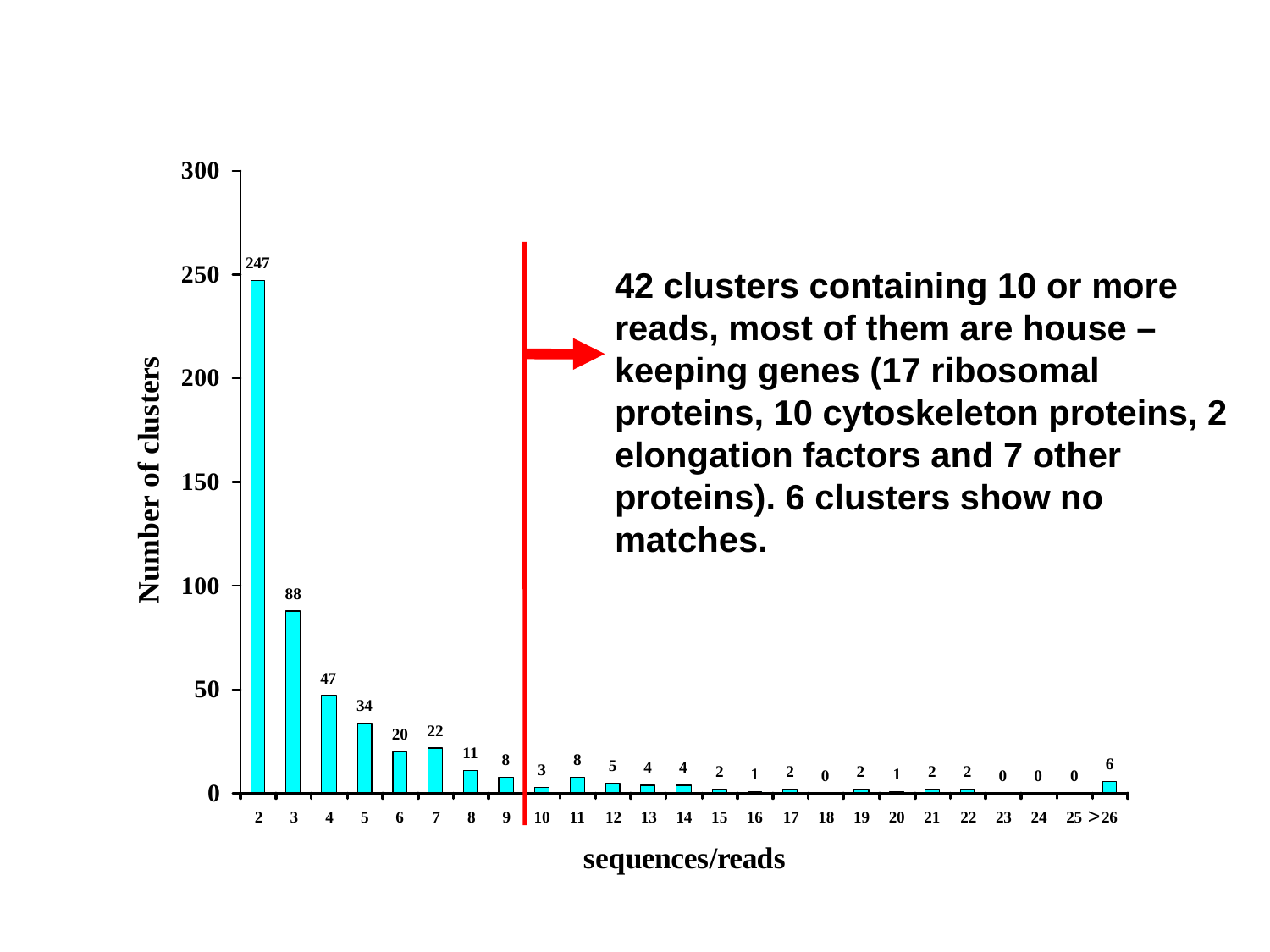

42 clusters containing 10 or more reads, most of them are house –keeping genes (17 ribosomal proteins, 10 cytoskeleton proteins, 2 elongation factors and 7 other proteins). 6 clusters show no matches.
>
