# Supplementary material for: An EST screen from the annelid Pomatoceros lamarckii reveals patterns of gene loss and gain in animals
Source: BMC Evol Biol. 2009 Sep 25;9:240. doi: 10.1186/1471-2148-9-240 (PMC2762978; doi:10.1186/1471-2148-9-240)
Supplement: Additional file 3 — A table of the evolutionary distance values in Figure 1A and 1B. The table shows the evolutionary distance values from the root where Nematostella and all bilaterian animal species diverge in Figure 1A and 1B. The exact probability values from the distance-based relative rate tests are also provided. The cells of the table are highlighted with red for significantly higher/longer distance values than that of Pomatoceros (P < 0.001) and green for lower/shorter values. [file 1471-2148-9-240-S3.DOC]

| Species\Tree | ML (1A) | NJ (1B) | probability |
| --- | --- | --- | --- |
| *Branchiostoma floridae* | 0.22904 | 0.13013 | 0.0001386 |
| *Homo sapiens* | 0.23694 | 0.13081 | 0.0005790 |
| *Ciona intestinalis* | 0.42188 | 0.20595 | 0.0000001> |
|  |  |  |  |
| *Lottia gigantea* | 0.37905 | 0.18376 | 0.0000014 |
| *Pomatoceros lamarckii* | 0.32615 | 0.15395 |  |
| *Capitella sp. I* | 0.32421 | 0.15593 | 0.7208740 |
|  |  |  |  |
| *Caenorhabditis elegans* | 0.56441 | 0.25113 | 0.0000001> |
| *Tribolium castaneum* | 0.32935 | 0.15732 | 0.1002830 |
| *Apis mellifera* | 0.38015 | 0.17560 | 0.0000502 |
| *Anopheles gambiae* | 0.40484 | 0.18401 | 0.0000017 |
| *Drosophila melanogaster* | 0.41174 | 0.18352 | 0.0000008 |
