# Supplementary material for: An EST screen from the annelid Pomatoceros lamarckii reveals patterns of gene loss and gain in animals
Source: BMC Evol Biol. 2009 Sep 25;9:240. doi: 10.1186/1471-2148-9-240 (PMC2762978; doi:10.1186/1471-2148-9-240)
Supplement: Additional file 8 — P. lamarckii protein sequences listed in Table 2. P. lamarckii predicted amino acid sequences in Table 2 are provided. The three proteins that are described in Figure 4 are not shown here. A. Pomatoceros -FVamide neuropeptide precursor protein sequence predicted from entire sequence of pl_xlvo_23a09 [GenBank: GQ381306]. RPRFV, RRMFV, RPKFV motifs are highlighted with blue. B. Pomatoceros galactose-binding lectin sequences predicted from pl_xlvo_33f01 and pl_xlvo_58g09 [GenBank: GQ381307]. The G-X-X-X-Q-X-W motifs are highlighted with yellow and related motif with blue. C. Pomatoceros FMRFamide precursor protein sequence predicted from Contig 196. FMRF motifs are highlighted with yellow. [file 1471-2148-9-240-S8.DOC]

**A** (pl_xlvo_23a09)

MTSQRFLFVLLYIAPLSFCEVMYPDLDPEEEAERNRVENLIEQYAMNELIADQLSAGLSASNDANEFQTADEHTNFQSLSKRPRFVGKRDQDEELDIGEFSKRRMFVGKRPMYVGKRNYFGDSVSSKRRMFVGKRDSMDSIDKRPRFVGKRFYMDDSDSLSNEVAKRPRFVGKRSYENDLLDFDKRRMFVGKKAILGQQYDKRPKFVGKRFMLYEDDLDKRSRNIFAKDFDKRPKFVGKRFEPSSDDDFNDLSFLYGEVEKRPRFVGKRRMFVGKRSEEDTSSFDKRRMFVGKRSAFDSTMTDDMPVEVNKNMFSDQSNEKRRMFVGKRDNEIADMDLFKNGGDLDSLVLNSNDKRSIDSPRDRLYTDDNLTTGLDKKAANRFVGKRSVNLKTAVEHARNKEGFLTAGRV

**B** (pl_xlvo_33f01)

LLINMSDDFPPLPKGKFFYIIGRESGLVLEINDANLEPGAPVVLGPKRDIRDEPVQSLCQMWYNCLVTGTIRCRANANLAFTVNDNDHICVDVVFEEEGRFFPTDRQRWFFNLKKGVIKSQEIDGRIRNMEVANHRTEPGTTVLTNEETGEDNQLFDAEYLDSPYFMLVSDMNGLCMEVDVGLFGGVKEGQKVVISKKDGKDNQLWYEDNFGVLRAKAGGYAIEWHRNLVRLCDAQSQRPSCNQHWHLS

(pl_xlvo_58g09)

MAEKKTPEGMYFVVRSRRNNNLVLDVLGGEMEAGKVCCMAEYNGSVSQIWYEDQVTSTIRSKSSDLCLIIGSDKILMVDEYKDKAEGQEWVLAKDKIQDNNNPKIVVEISDANGEVDAQLTQGELKNEPHQLFDIDYQDAVYFYIVSELHGKVVTVKHAETRPDAKIVIEPKREGACEQLWHEGKHGFMRSKLNNFVLEAKENRNGASMRLMPFEPGNSKQLWCRHHGKILSLVHPKDILEIKKKKKDNGAKLVIGDDNNLPNQTWIFEEVSSE

**C** (Contig 196)

SDPDPDVYNNENGEKRFMRFGRAGNNDDPSESGEKRFMRFGKRANMKPLDIEDIDPNKRFMRFGKRNQNGYDQIESKTHRGKRSIREDGQKNSETDTSHADKGSNFKGTNESVDDIMGTINGLDAIDKVTKSSDTEKRSKSGYEKKLNRMFPKTKKFMRFGKRGFELNQENMEGLEMAKLTIEDLENHFSKVMKRQDEILEPIHIPSRWNKLI
